# Tissue‐specific methylomic responses to a lifestyle intervention in older adults associate with metabolic and physiological health improvements

**DOI:** 10.1111/acel.14431

**Published:** 2024-12-01

**Authors:** Lucy Sinke, Marian Beekman, Yotam Raz, Thies Gehrmann, Ioannis Moustakas, Alexis Boulinguiez, Nico Lakenberg, Eka Suchiman, Fatih A. Bogaards, Daniele Bizzarri, Erik B. van den Akker, Melanie Waldenberger, Gillian Butler‐Browne, Capucine Trollet, C. P. G. M. de Groot, Bastiaan T. Heijmans, P. Eline Slagboom

**Affiliations:** ^1^ Molecular Epidemiology, Department of Biomedical Data Sciences Leiden University Medical Centre Leiden The Netherlands; ^2^ Department of Bioscience Engineering, Research Group Environmental Ecology and Applied Microbiology University of Antwerp Antwerp Belgium; ^3^ Sequencing Analysis Support Core, Department of Biomedical Data Sciences Leiden University Medical Center Leiden The Netherlands; ^4^ Myology Center for Research, U974 Sorbonne Université, INSERM, AIM, GH Pitié Salpêtrière Bat Babinski Paris France; ^5^ Division of Human Nutrition Wageningen University and Research Wageningen The Netherlands; ^6^ Delft Bioinformatics Lab, Pattern Recognition and Bioinformatics Delft The Netherlands; ^7^ Research Unit Molecular Epidemiology, Institute of Epidemiology Helmholtz Munich, German Research Center for Environmental Health Neuherberg Germany; ^8^ German Center for Cardiovascular Research (DZHK) Partner Site Munich Heart Alliance Munich Germany

**Keywords:** DNA methylation, epigenomics, functional genomics, healthy aging, lifestyle, metabolism, muscle

## Abstract

Across the lifespan, diet and physical activity profiles substantially influence immunometabolic health. DNA methylation, as a tissue‐specific marker sensitive to behavioral change, may mediate these effects through modulation of transcription factor binding and subsequent gene expression. Despite this, few human studies have profiled DNA methylation and gene expression simultaneously in multiple tissues or examined how molecular levels react and interact in response to lifestyle changes. The Growing Old Together (GOTO) study is a 13‐week lifestyle intervention in older adults, which imparted health benefits to participants. Here, we characterize the DNA methylation response to this intervention at over 750 thousand CpGs in muscle, adipose, and blood. Differentially methylated sites are enriched for active chromatin states, located close to relevant transcription factor binding sites, and associated with changing expression of insulin sensitivity genes and health parameters. In addition, measures of biological age are consistently reduced, with decreases in grimAge associated with observed health improvements. Taken together, our results identify responsive molecular markers and demonstrate their potential to measure progression and finetune treatment of age‐related risks and diseases.

## Graphical abstract

Diet and physical activity influence immunometabolic health across the lifespan, and DNA methylation may mediate a part of these effects. The Growing Old Together (GOTO) study, a 13‐week lifestyle intervention in older adults, revealed epigenetic changes in muscle, adipose, and blood at hundreds of CpGs, linking these to differential gene expression, health improvements, and finding that the intervention consistently decreased participants' biological age.

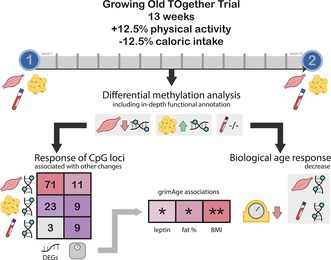

**Abbreviations**
bAge: biological age
BMI: body mass index
cAge: chronological age
CALERIE: Comprehensive Assessment of Long term Effects of Reducing Intake of Energy
DEG: differentially expressed gene
DMP: differentially methylated probe
DMR: differentially methylated region
DNAm: DNA methylation
FDR: false discovery rate
FFQ: food frequency questionnaire
GO: gene ontology
GOTO: Growing Old Together
GSEA: gene set enrichment analysis
HDL: high‐density lipoprotein
IL‐6: interleukin‐6
LLS: Leiden Longevity Study
MuSiC: Multi‐subject Single‐cell deconvolution
MVEC: microvascular endothelial cell
OR: odds ratio
PC: principal component
PCA: principal component analysis
QC: quality control
SAT: subcutaneous adipose tissue
SBP: systolic blood pressure
TF: transcription factor
TFBS: transcription factor binding site
TSS: transcription start site
WC: waist circumference

## 1 INTRODUCTION

Changes in behavior across the life course, including adherence to healthy diets and physical activity, have major health impacts and in some cases are more effective at improving immunometabolic health than pharmacological interventions (Diabetes Prevention Program (DPP) Research Group, 2002; Knowler et al., 2002; Fiuza‐Luces et al., 2013). Regular aerobic exercise alongside caloric restriction promotes weight loss, insulin sensitivity, and glucose control in both younger and older populations (Eriksson et al., 1999; Kraus et al., 2019; Leitner et al., 2017; Zhang et al., 2020). Epigenetic regulation, such as through DNA methylation (DNAm), may mediate a part of these health benefits by modulating the accessibility of regulatory sites for transcription factors (Gevaert et al., 2022; van der Harst et al., 2017; Voisin et al., 2015). Physical activity has been found to attenuate the age‐dependent decreases in DNAm of the anti‐inflammatory *ASC* gene in blood (Butts et al., 2018; Nakajima et al., 2010), and Mendelian randomization has directionally linked epigenetic signatures of a healthy diet with both type 2 diabetes and several of its risk factors (Ma et al., 2020). These findings highlight the potential impact of DNAm for measurement and modification of immunometabolic health in individuals of all ages.

In comparison to findings in blood, epigenetic reprogramming in metabolic tissues may have even greater functional consequences on health, but studies that collect tissues other than blood in sufficient sample sizes are sparse (King‐Himmelreich et al., 2016). Muscle and adipose tissues are known to secrete a plethora of proteins and signalling molecules into the circulation and engage in tissue‐to‐tissue crosstalk, collectively bringing about biological changes (Stanford & Goodyear, 2018). Although several experimental studies have investigated the effects of lifestyle interventions on the methylome of muscle (Barrès et al., 2012; Jacobsen et al., 2012) or adipose tissues (Fabre et al., 2018; Gillberg et al., 2014; Rönn et al., 2013) individually, few researchers have taken a multi‐tissue approach despite this showing promise in other‐omic fields (Mill & Heijmans, 2013; Moore et al., 2023; Savikj et al., 2022). As we advance our understanding of how epigenetics influences immunometabolic health, it is important to diversify our studies to incorporate relevant tissues, improve CpG coverage, and be inclusive of older adults who represent a growing proportion of our populations.

The Growing Old Together (GOTO) study is a 13‐week lifestyle intervention in 164 older adults (mean age 63 years), which expanded on the combined intervention arm of the CALERIE study (Rickman et al., 2011). Here, we followed up on previous work showing that the GOTO intervention conferred an improvement in immunometabolic health (Beekman et al., 2020; van de Rest et al., 2016) and that this benefit associates with changes in the blood (Gehrmann et al., 2021), adipose, and muscle transcriptomes (Bogaards et al., 2024) and the blood metabolome (Bogaards et al., 2022). Using data and biomaterial from before and after the GOTO study, we profiled DNAm at over 750 thousand CpG sites across the genome in skeletal muscle (*n* = 80), subcutaneous adipose (*n* = 89), and fasted blood tissues (*n* = 98). By thoroughly characterizing the resulting loci, we examined how methylomic responses to the GOTO intervention related to genomic regulation and differential gene expression in *cis*, with implications for immunometabolic health and epigenetic measures of chronological and biological age.

## 2 RESULTS

### 2.1 The GOTO intervention improves metabolic health, which is consistently observed in the tissue‐dependent subsets of participants

The Growing Old Together (GOTO) intervention (*n* = 164) imparted a range of metabolic health benefits, described in detail previously (Bogaards et al., 2022, 2024; Gehrmann et al., 2021; van de Rest et al., 2016). Notably, participants saw reductions in their body mass index (BMI, Δ = −1.1 kg/m (Knowler et al., 2002)), waist circumference (WC, Δ = −4.3 cm), and total body fat percentage (Δ = −1.8%) alongside improvements in many other health measurements (Table 1, Table S1). Individuals were selected for DNA methylation profiling based on availability of biological material and gene expression data, and for a majority (*n* = 66, 64.7%), we were able to collect data from all three tissues both before and after the GOTO intervention.

**TABLE 1:** Effects of the 13‐week GOTO intervention on 10 metabolic health measurements in the entire population and each tissue‐dependent subset.

	Entire GOTO population			Three tissue overlap			Skeletal muscle tissue			Subcutaneous adipose			Fasted blood		
	(*n* = 164)			(*n* = 66)			(*n* = 80)			(*n* = 89)			(*n* = 98)		
	Δ	SE	*p*_FDR_	Δ	SE	*p*_FDR_	Δ	SE	*p*_FDR_	Δ	SE	p_FDR_	Δ	SE	*p*_FDR_
BMI (kg/m^2^)	−1.13	0.06	2.0 × 10^−39^	−1.24	0.10	1.2 × 10^−17^	−1.28	0.09	4.2 × 10^−23^	−1.27	0.08	1.5 × 10^−26^	−1.28	0.08	1.9 × 10^−29^
WC (cm)	−4.32	0.42	5.6 × 10^−19^	−4.83	0.66	2.7 × 10^−9^	−5.10	0.60	5.2 × 10^−12^	−4.53	0.58	5.6 × 10^−11^	−4.65	0.55	9.8 × 10^−13^
Total body fat (%)	−1.76	0.23	8.7 × 10^−12^	−2.13	0.35	2.3 × 10^−7^	−2.03	0.34	9.4 × 10^−8^	−2.18	0.30	2.7 × 10^−10^	−2.05	0.30	1.9 × 10^−9^
Fasting Insulin (mU/L)	−0.31	0.25	2.7 × 10^−1^	−0.83	0.41	5.8 × 10^−2^	−0.55	0.38	1.7 × 10^−1^	−0.45	0.38	2.7 × 10^−1^	−0.46	0.34	2.0 × 10^−1^
SBP (mm Hg)	−3.15	0.94	1.6 × 10^−3^	−2.82	1.68	1.1 × 10^−1^	−2.66	1.46	1.0 × 10^−1^	−2.88	1.34	4.9 × 10^−2^	−2.51	1.25	6.8 × 10^−2^
Leptin (ug/L)	−2.32	0.34	3.7 × 10^−10^	−2.75	0.44	1.4 × 10^−7^	−2.72	0.38	9.4 × 10^−10^	−2.95	0.40	2.7 × 10^−10^	−2.86	0.37	1.9 × 10^−11^
Adiponectin (mg/L)	0.26	0.14	1.1 × 10^−1^	0.52	0.25	5.8 × 10^−2^	0.45	0.21	6.1 × 10^−2^	0.27	0.21	2.5 × 10^−1^	0.28	0.20	2.0 × 10^−1^
IL‐6 (ng/L)	0.09	0.11	4.8 × 10^−1^	0.30	0.15	5.8 × 10^−2^	0.23	0.13	1.1 × 10^−1^	0.24	0.11	4.9 × 10^−2^	0.26	0.12	5.8 × 10^−2^
Fasting HDL size (nm)	0.04	0.01	5.7 × 10^−9^	0.06	0.01	1.3 × 10^−5^	0.07	0.01	1.9 × 10^−8^	0.05	0.01	6.6 × 10^−7^	0.05	0.01	3.0 × 10^−8^
HDL‐C (mmol/L)	−0.01	0.02	6.2 × 10^−1^	0.00	0.03	9.3 × 10^−1^	0.00	0.03	9.4 × 10^−1^	−0.02	0.02	5.3 × 10^−1^	−0.02	0.02	4.8 × 10^−1^

*Note*: Associations were calculated from paired data using linear mixed models with fixed effects for age and sex and a random effect for ID. P‐values were adjusted using the FDR correction method.

Abbreviations: BMI, body mass index; HDL‐C, high density lipoprotein cholesterol; IL‐6, interleukin‐6; SBP, systolic blood pressure; WC, waist circumference.

For each tissue, methylation subsets were representative of the whole study population (muscle *n* = 80, SAT *n* = 89, and blood *n* = 98), with the distribution of changes in 10 health parameters from included and excluded individuals being statistically comparable (nonresponse analysis *p*_FDR_ > 0.05, Table S2). The sole exception was a selection bias for individuals with higher HDL sizes in the muscle tissue subset (*p*_FDR_ = 0.001) urging caution in making inferences about this trait in muscle. Each subset was analysed for genome‐wide DNAm consequences of the GOTO intervention, adjusting for age, sex, smoking status, technical covariates, and the first five principal components (PCs; see Section 5). In the skeletal muscle and SAT samples, estimated bias and inflation of the test statistics was low (|μ| < 0.05, λ < 1.1). In the blood samples, there was some deflation in the test statistics (λ = 0.86) alongside minimal bias (μ = 0.03). For all tissues, corrected bias and inflation was under 0.01 and equal to 1.0 respectively, indicating high quality data.

### 2.2 In skeletal muscle, the GOTO intervention influenced DNA methylation at 162 predominantly hypomethylated CpGs

**FIGURE 1:**
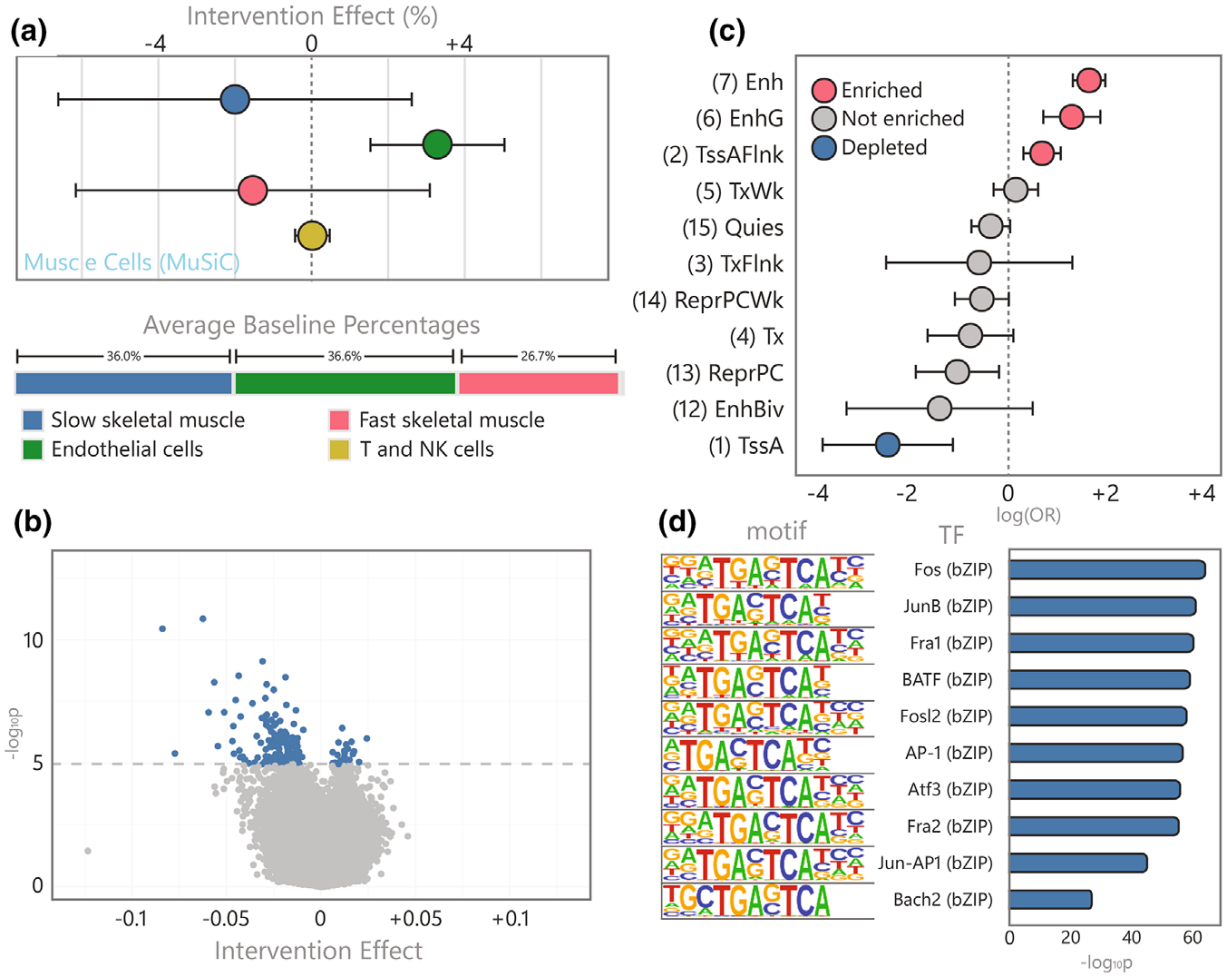
Characterization of the muscle cell count and DNA methylation response to the GOTO intervention (a) Intervention effect on muscle cell types predicted using MuSiC alongside baseline proportions (only cells >0.5% at baseline shown), (b) Volcano plot of the intervention effect on DNAm at over 750 thousand CpGs, showing the 162 significant CpGs in blue and nonsignificant in grey, (c) Forest plots showing the OR and 95% CI for enrichment or depletion of 11 ROADMAP chromatin states in the 162 muscle CpGs using the E107 male skeletal muscle reference epigenome (four states with extremely wide CIs not shown), and (d) Bar plot of the top 10 enriched TFBS motifs in sequences within 50 bp of the 162 muscle CpGs.

To record the response of the muscle methylome, we profiled DNAm in skeletal muscle samples biopsied before and after the GOTO intervention (*n* = 160 samples, 80 individuals). Since cell‐type proportions can be an important driver of epigenetic signals, we predicted proportions of seven muscle nuclei types in our samples by applying the MuSiC algorithm (Wang et al., 2019) to bulk gene expression data (Bogaards et al., 2024) and a publicly available single nuclei transcriptomic reference (Perez et al., 2022). At baseline, our samples were predicted to primarily be composed of slow (type I, mean 36.0%) and fast (type II, mean 26.7%) skeletal muscle fibres and endothelial cells (mean 36.6%). Following the intervention, there was evidence that the proportion of predicted endothelial nuclei in the muscle tissue had increased (Δ = +3.3%, *p*_FDR_ = 3.5 × 10^−3^), in line with expected angiogenesis during the intervention (Kwak et al., 2018), and there was insufficient evidence for changes in any of the other nuclei types (*p*_FDR_ > 0.05; Figure 1a, Table S3).

In our initial model, we identified 354 CpGs differentially methylated following the intervention (*p*_FDR_ <0.05). However, considering the finding that an increase in endothelial nuclei could have been driving a portion of this methylation signal, we further adjusted our model for predicted endothelial nuclei proportions. This led to the removal of 192 CpGs from our results, leaving 162 predominantly hypomethylated (87.7%) CpGs where DNAm changes were independent of endothelial nuclei proportions (*p*_FDR_ ≤ 0.05; Figure 1b, Table S4). Henceforth, we refer to this set of 162 differentially methylated CpGs in skeletal muscle, which represent 160 distinct loci, as the *muscle CpGs*.

### 2.3 CpGs influenced by the GOTO intervention associate with genes important for translocation of GLUT4 to the muscle cell membrane

To investigate the functional capacity of these *muscle CpGs* to influence nearby gene expression, we annotated their genomic positions to 15 chromatin states using Roadmap reference epigenomes (Kundaje et al., 2015). These consist of eight active and seven repressed states that show distinct levels of DNA methylation, accessibility, and regulator binding. By testing if the *muscle CpGs* were enriched for any particular genomic feature in the male (E107) and female (E108) skeletal muscle reference, we revealed that both enhancers (OR_E107_ = 5.83 and OR_E108_ = 7.15) and genic enhancers (OR_E107_ = 3.56 and OR_E108_ = 4.34) were overrepresented in our results (Figure 1c, Table S5). In addition, since the primary mechanism that DNAm influences nearby expression is through transcription factor (TF) binding (Kaluscha et al., 2022), we also tested if sequences within 50 bp of the *muscle CpGs* were enriched for known TF binding sites (TFBS; Figure 1d; Table S6). The tested regions were enriched for 21 TFBS including ones known to be both upregulated by exercise (JunB: 34 CpGs) (Trenerry et al., 2007) and critical for muscle regeneration (Fos: 35 CpGs; Fra1: 33 CpGs) (Almada et al., 2021; Galvagni et al., 2002; Puntschart et al., 1998). Taken together, these functional analyses support this set of 162 CpGs as located at *cis* regulatory regions specifically in skeletal muscle cells.

After establishing the *muscle CpGs* as plausibly regulatory, we identified nearby genes that were candidate targets for this regulation using a two‐step approach. Firstly, we evaluated if the expression of genes in close proximity (±100 kb) to the 162 *muscle CpGs* was altered by the intervention using gene expression data previously collected from this study (*p*_FDR_ ≤ 0.05) (Bogaards et al., 2024). Secondly, we examined whether these gene expression changes were associated with differential DNAm at the nearby CpG (*p*_FDR_ ≤ 0.05). There were 454 unique genes within 100 kb of a *muscle CpG*, and 71 of these were both differentially expressed following the intervention and associated with DNAm *in cis* (Table S7). This set of 71 genes included several directly implicated in the translocation of GLUT4 transporters to the muscle cell membrane in response to insulin and contractile activity (*TMOD3*, *FDFT1*, and *PLEKHG4*) (Ha & Lee, 2020; Machin et al., 2021; Shrestha et al., 2021) alongside an adaptor protein which regulates insulin signalling, including specifically in skeletal muscle cells (*GRB10*) (Edick et al., 2020; Holt et al., 2018). Gene set enrichment analysis of these 71 genes also revealed enrichment for Striated Muscle Cell Development (*p*adj = 0.019) after adjusting for multiple testing, further clarifying the overrepresentation of muscle‐related genes in this set (Table S8).

### 2.4 Altered blood‐based health markers and grip strength associate with DNA methylation responses in skeletal muscle

**FIGURE 2:**
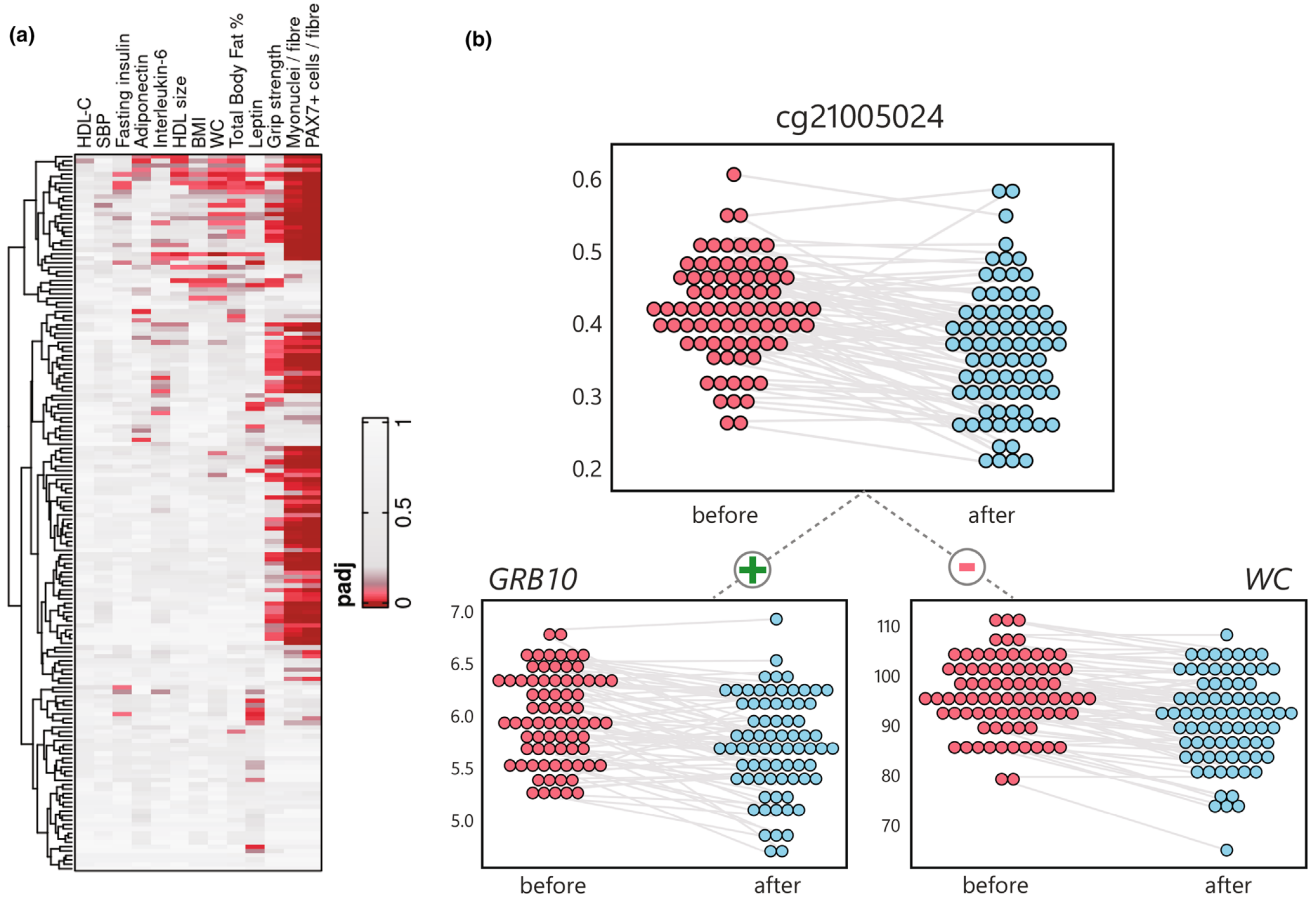
Multi‐omic analyses in muscle (a) Clustered heatmap of association *p* values between changes in DNAm at the 162 muscle CpGs and 10 health parameters (BMI, body mass index; HDL, high‐density lipoprotein; IL‐6, inetrleukin‐6; SBP, systolic blood pressure; WC, waist circumference) and (b) Dot plots showing hypomethylation of DNAm in muscle at cg21005024, positively associated with decreases in muscle *GRB10* gene expression and in waist circumference.

Next, we investigated whether the observed methylomic responses to the GOTO intervention in muscle associated with changes in blood‐based measures of metabolic health. We performed paired analyses of the associations between DNAm effects and improvements in the 10 health parameters shown in Table 1, adjusting for multiple testing using the FDR method (Table S9, Figure 2a). DNAm at 33 (20.4%) of the *muscle CpGs* associated with at least one blood‐based trait, with eight CpGs being linked to improvements in three or more traits (*p*_FDR_ ≤ 0.05).

We also investigated how effects on the muscle methylome related to changes in biopsy‐specific muscle physiology, represented by number of PAX7‐positive cells and myonuclei per fibre as obtained from previous immunohistochemistry work (*n* = 65, 81% of the original muscle subset) (Raz et al., 2020), and overall muscle performance as measured by average dominant hand grip strength. PAX7 is a satellite cell marker and, as the number of these per fibre increases, the regenerative potential of the muscle is also higher (Azhar et al., 2022). Higher numbers of myonuclei per fibre suggests larger and stronger muscle fibres (Hansson et al., 2020; Snijders et al., 2021). Both immunohistochemistry measures were associated with DNAm responses at over 70 differentially methylated CpGs (N_MYO_ = 75, N_PAX7_ = 84), and DNAm at 36 CpGs was associated with increasing average dominant hand grip strength (Table S10). These CpGs largely included those found to be associated with improvements in blood‐based health markers. In total, well over half of the *muscle CpGs* (*N* = 103, 61.7%) were associated with at least one of the investigated health traits, demonstrating the importance of this set of CpGs to observed health improvements.

More specifically, there were 16 CpGs that were differentially methylated, located in *cis* regulatory regions, and also associated with both differential gene expression and observed health benefits. One example of these CpGs that highlights the potential relevance of our findings in skeletal muscle is *cg21005024*, whose methylation decreased following the GOTO intervention (*β* = −0.047). This CpG flanks an active transcription start site (TSS) of *GRB10* within 50 bp of multiple enriched TFBS. Hypomethylation of *cg21005024* is associated with decreasing *GRB10* expression (*β* = 0.083), waist circumference (*β* = 0.003), and total body fat percentage (*β* = 0.005) following the intervention, as well as increases in both immunohistochemistry measures (*β*_PAX7_ = −0.220, *β*_MYO_ = −0.011) and average dominant hand grip strength (*β* = −0.004; Figure 2b).

### 2.5 In adipose tissue, the GOTO intervention influenced DNA methylation at 230 predominantly hypermethylated CpGs

**FIGURE 3:**
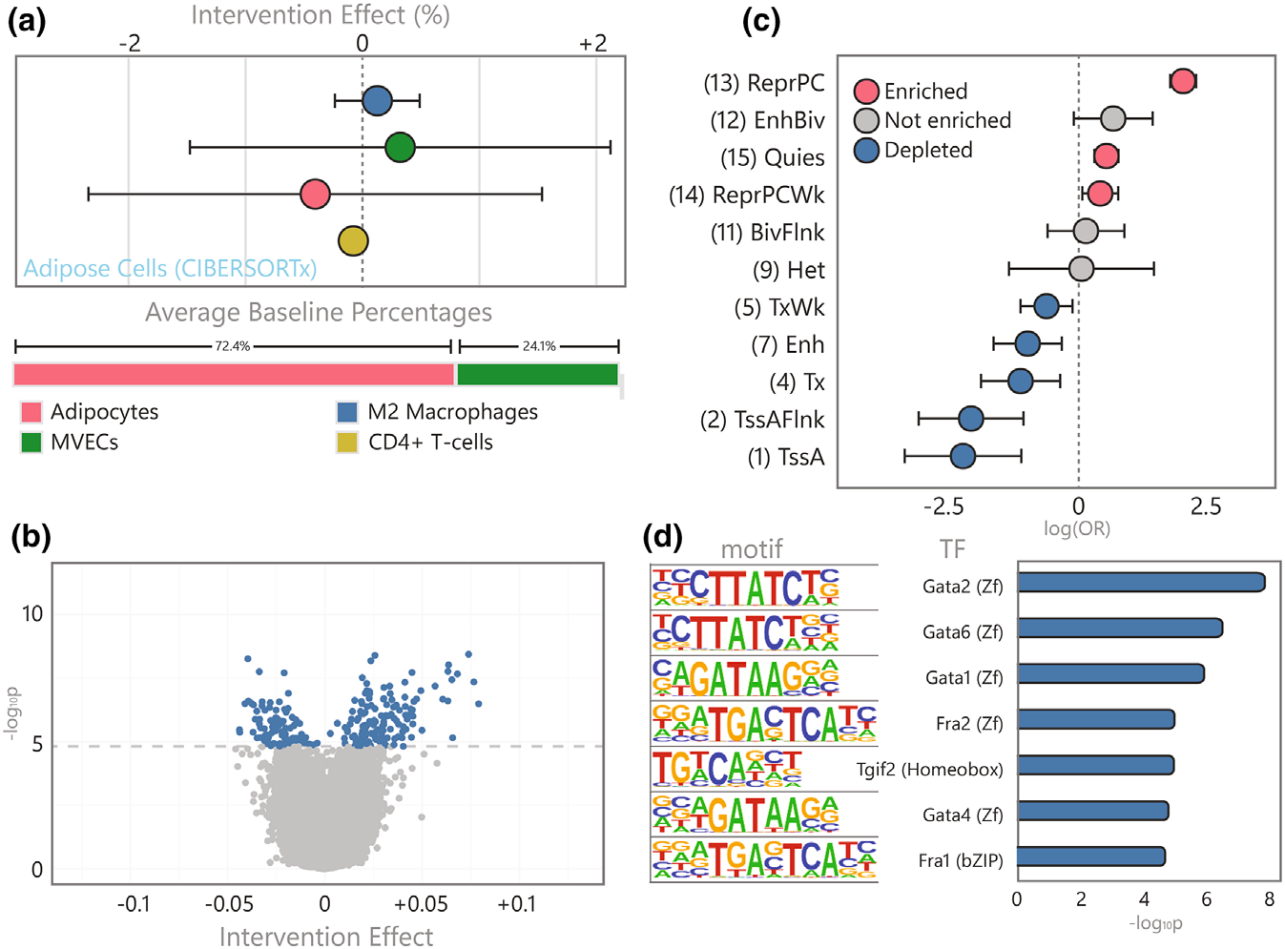
Characterization of the adipose cell count and DNA methylation response to the GOTO intervention (a) Intervention effect on adipose cell types predicted using CIBERSORTx alongside baseline proportions (only cells >0.5% at baseline shown), (b) Volcano plot of the intervention effect on DNAm at over 750 thousand CpGs, showing the 230 significant CpGs in blue and nonsignificant in grey, (c) Forest plots showing the OR and 95% CI for enrichment or depletion of 11 ROADMAP chromatin states in the 230 adipose CpGs using the E063 adipose reference epigenome (four states with extremely wide CIs not shown), and (d) Bar plot of the seven enriched TFBS motifs in sequences within 50 bp of the 230 adipose CpGs.

Next, we investigated DNAm changes in the subcutaneous adipose tissue (SAT) following the GOTO intervention (*n* = 89 individuals, 178 samples). We identified 230 differentially methylated CpGs at 201 distinct loci (*p*_FDR_ ≤ 0.05), henceforth referred to as the *adipose CpGs*. To explore whether this methylation signal was driven by changes in cellular tissue composition, we predicted five cell‐type proportions from bulk gene expression data using the CIBERSORTx algorithm (Newman et al., 2019) and a publicly available signature matrix (Glastonbury et al., 2019) (Figure 3a, Table S11). The most prevalent cells at baseline were adipocytes (mean 72.4%) followed by a large minority of microvascular endothelial cells (MVECs; mean 24.1%). However, since there was insufficient evidence to support a change in any of these predicted cell types following the intervention (*p*_FDR_ >0.05), we did not consider further adjustment appropriate in our paired analyses investigating differential methylation in SAT.

### 2.6 CpGs influenced by the GOTO intervention associated with genes linked to lipid metabolism and insulin resistance

The majority (60.4%) of the 230 *adipose CpGs* were hypermethylated following the intervention (Figure 3b, Table S12) and, to determine whether this represented a plausibly functional signal, we annotated their genomic positions to 15 chromatin states using the Roadmap adipose reference epigenome (E063) (Kundaje et al., 2015). Since the *adipose CpGs* were enriched for several repressive marks, such as polycomb repressed regions (OR = 7.76, *p*_FDR_ = 7.35 × 10^−43^) and depleted for regulatory states, like enhancers (OR = 0.38, *p*_FDR_ = 1.10 × 10^−2^) and active TSS (OR = 0.11, *p*_FDR_ = 5.21 × 10^−4^), there was insufficient evidence to suggest that the *adipose CpGs* as a whole were controlling nearby transcription (Figure 3c, Table S13). Despite this, sequences within 50 bp of the *adipose CpGs* were enriched for seven known TFBS (Figure 3d, Table S14), including four GATA family TFBS whose associated TFs are involved in the initial stages of adipogenesis and obesity (Tong et al., 2000).

Although the *adipose CpGs* did not likely represent a regulatory set overall, we explored whether they were individually associated with changes in expression of nearby genes (±100 kb). We first investigated if genes in *cis* were differentially expressed (DEGs) following the GOTO intervention and, if so, whether this change in gene expression was associated with altered methylation at the nearby CpG. Within 100 kb of the 230 *adipose CpGs* there were 412 genes, and for 23 of these there was both evidence that expression changed as a result of the intervention (*p*_FDR_ ≤ 0.05) and that this change was associated with the nearby DNAm response (*p*_FDR_ ≤ 0.05, Table S15).

These 23 genes included many relevant for adipogenesis, such as *ZBTB7A* (Laudes et al., 2004) and *ALX1* (Breitfeld et al., 2020) and multiple developmental genes including *EN1* and *NR2F1* (Singh et al., 2015). Of particular interest were *PITX2* and *DMRT3*, whose decreasing expressions were associated with responses at 3 and 16 unique *adipose CpGs*, respectively. *PITX2* encodes a transcription factor which has been linked to changes in fasting glucose following weight loss (Macartney‐Coxson et al., 2017) and *DMRT3*, which associates with exercise training and diet (Divoux et al., 2021; Nono Nankam et al., 2020), has been proposed as a marker of insulin resistance specifically in SAT (Clemente‐Olivo et al., 2021). To further investigate the importance of this set of 23 genes, we performed GSEA using clusterProfiler (Table S16). This revealed enrichment for 27 terms, including many relevant for lipid metabolism and transport (e.g., Phospholipid Efflux *p*_FDR_ = 0.019), as well as transcription (e.g., Regulation of Transcription by RNA Polymerase II *p*_FDR_ = 0.019).

### 2.7 Reductions in total body fat percentage associate with DNA methylation responses in adipose tissue

**FIGURE 4:**
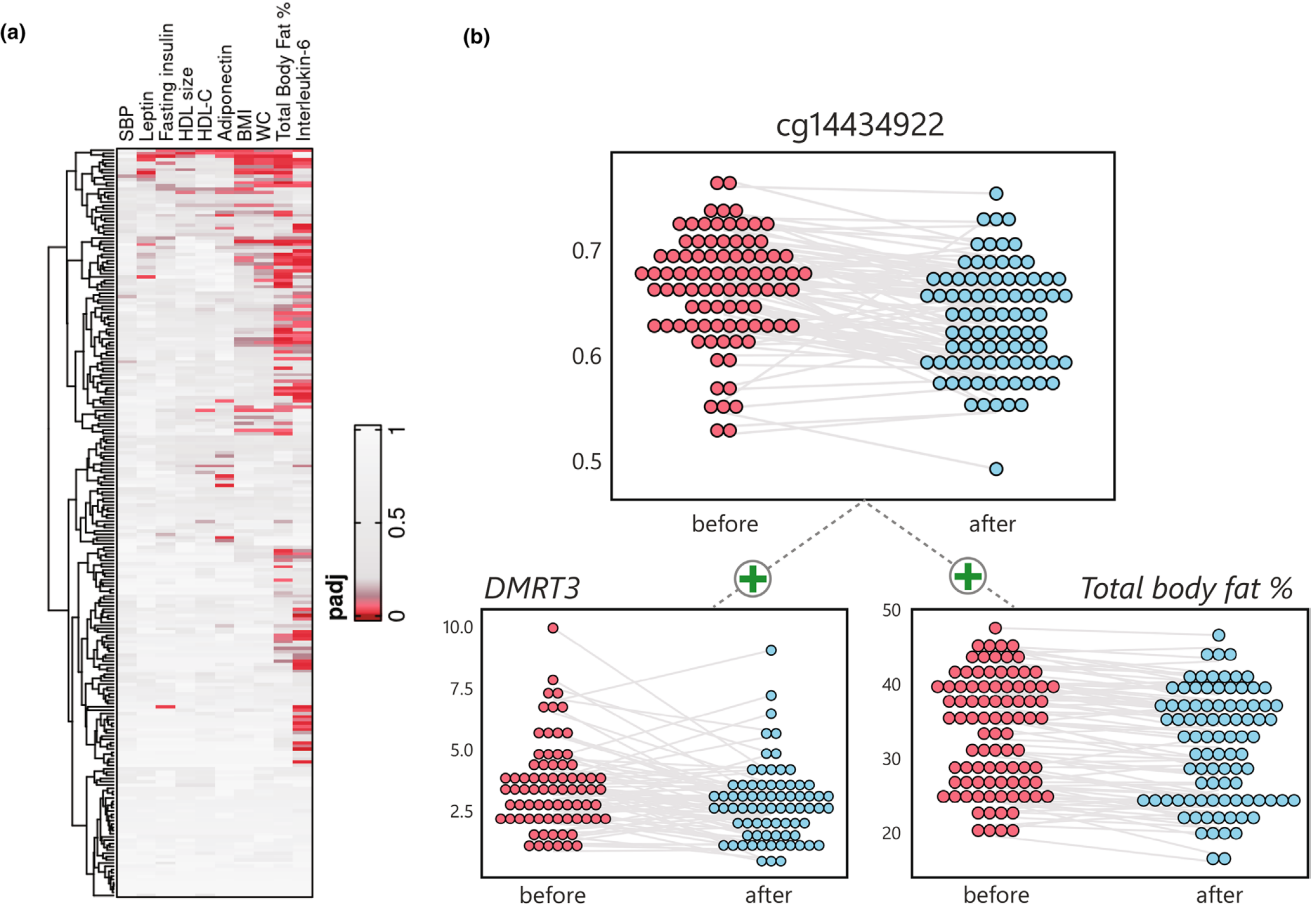
Multi‐omic analyses in adipose (a) Clustered heatmap of association *p* values between changes in DNAm at the 230 adipose CpGs and 10 health parameters (BMI, body mass index; HDL, high‐density lipoprotein; IL‐6, inetrleukin‐6; SBP, systolic blood pressure; WC, waist circumference) and (b) Dot plots showing hypomethylation of DNAm in muscle at cg14434922, positively associated with decreases in adipose *DMRT3* gene expression and in total body fat percentage.

To link the epigenetic findings in SAT to changes in health, we performed paired analyses of the association between DNAm effects at the *adipose CpGs* and changes in the 10 health parameters shown in Table 1 (Figure 4a, Table S17). In total, almost a third of the *adipose CpGs* (*N* = 75, 32.6%) were associated with improvements in at least one tested trait, and responses at more than five unique CpGs were associated with total body fat percentage (39 CpGs), adipocytokine levels (adiponectin: 8 CpGs; interleukin 6: 48 CpGs), BMI (12 CpGs), and WC (12 CpGs). Of these, 12 had been linked with nearby gene expression in the previous analyses. Indeed, hypomethylation at two of the three *DMRT3* associated *adipose CpGs* was associated with reductions in total body fat percentage (Figure 4b). Notably, these results link DNAm effects at multiple CpGs, differential expression of relevant genes, and improvements in health, indicating the relevance of the identified loci for physiological and molecular responses to lifestyle changes in older adults.

### 2.8 Altered blood‐based health parameters associate with the relatively small DNA methylation responses in blood

**FIGURE 5:**
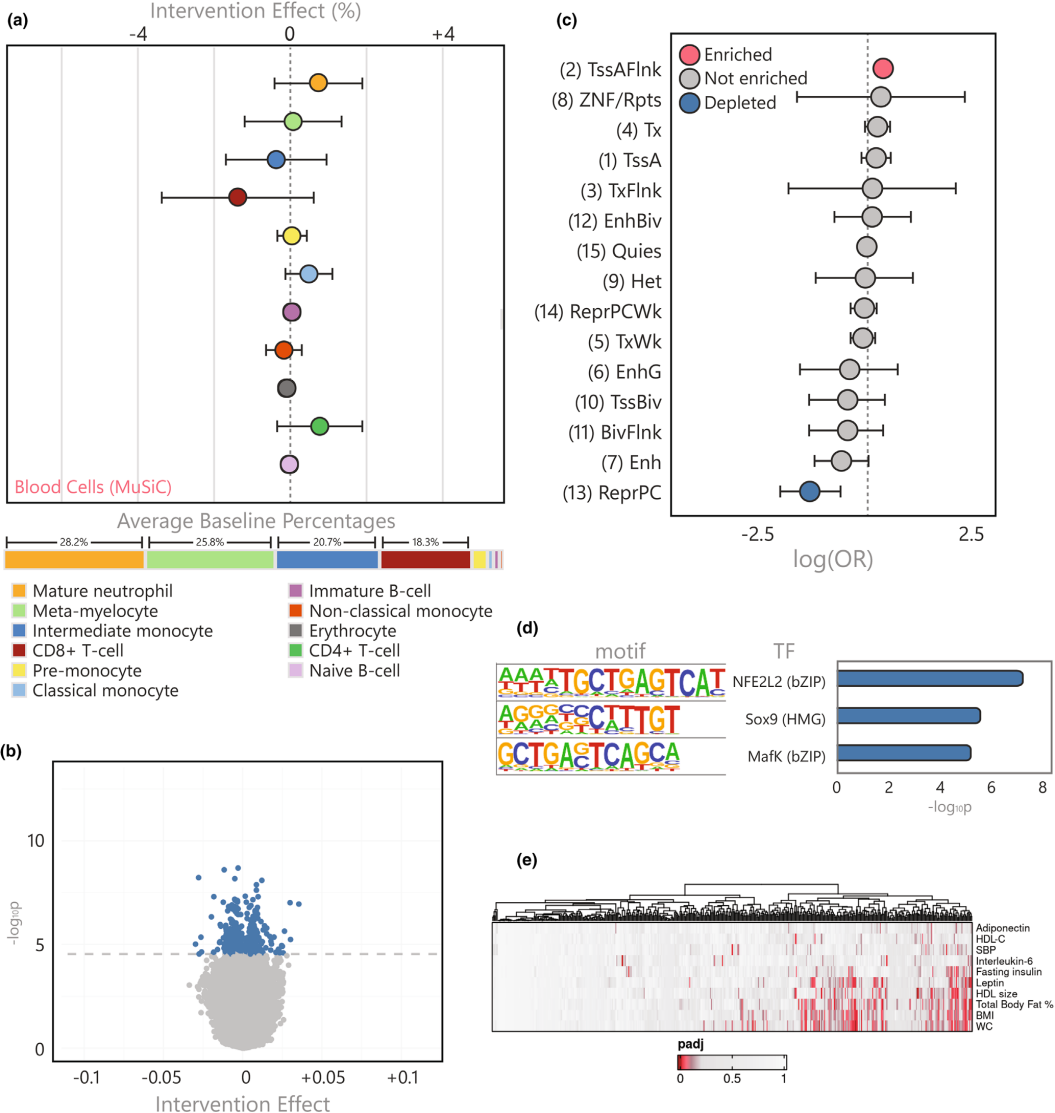
Characterization of the blood cell count and DNA methylation response to the GOTO intervention (a) Intervention effect on immune cell types predicted using the IDOL algorithm alongside baseline proportions (only cells >0.5% at baseline shown), (b) Volcano plot of the intervention effect on DNAm at over 750 thousand CpGs, showing the 441 significant CpGs in blue and nonsignificant in grey, (c) Forest plots showing the OR and 95% CI for enrichment or depletion of 15 ROADMAP chromatin states in the 441 blood CpGs using the E062 PBMC reference epigenome, (d) Bar plot of enriched TFBS motifs in sequences within 50 bp of the 441 blood CpGs, and (e) Clustered heatmap of association *p* values between changes in DNAm at the 441 blood CpGs and 10 health parameters (BMI, body mass index; HDL, high‐density lipoprotein; IL‐6, inetrleukin‐6; SBP, systolic blood pressure; WC, waist circumference).

Next, we analysed individuals with DNAm data from paired fasted blood samples (*N* = 98 individuals, 196 samples) and identified 441 CpGs at distinct loci where methylation was altered following the intervention (*p*_FDR_ ≤ 0.05), henceforth referred to as the *blood CpGs*. In cross‐sectional blood‐based EWAS, cell‐type proportions are a well‐known driver of association signals, and therefore we extensively assessed if changes in cell types not captured by the individual‐level random effects existed in our data. We measured five cell types and predicted a further 36 from DNAm and gene expression data. Neutrophils and their progenitors accounted for the majority of cells (between 47.7% and 51.8% of cells at baseline), followed by lymphocytes (36.9% to 41.1%) and then monocytes (7.4% to 10.1%). There was insufficient evidence to suggest any of the measured or predicted nucleated cell‐types changed following the intervention at either the 5% nominal or FDR level, indicating that cell‐type proportions were captured within the individual random effects in the models and additional adjustment would be redundant (Figure 5a; Table S18).

In contrast to the findings in skeletal muscle and SAT, all effect sizes at the 441 *blood CpGs* were small (*β* < 4%; Figure 5b, Table S19), but this alone did not preclude them from functionality. To investigate the likelihood that changes in DNAm at the *blood CpGs* were regulatory, we performed chromatin state and TFBS enrichment analyses as before. The *blood CpGs* were enriched (Figure 2c, Table S20) for regions flanking active TSS (OR: 1.50, p_FDR_ = 1.1 × 10^−2^), although the size of this enrichment was less convincing than seen in previous tissues. Sequences within 50 bp of the *blood CpGs* were enriched for three known TFBS (Figure 5d, Table S21), including NFE2L2 and MafK, which are involved in oxidative stress responses (Hwang et al., 2013; Ryoo & Kwak, 2018). When we investigated genes within 100 kb of the *blood CpGs*, there were only three genes both differentially expressed and linked to nearby methylation (Table S22), but these included ones critical for inflammatory and immune responses such as *LTBR* and *TNFRSF1A* (McDermott, 2001; Piao et al., 2021).

To explore possible distant or pleiotropic effects of the DNAm responses in blood on health, we performed paired analyses of the association between DNAm at the *blood CpGs* and changes in the 10 health parameters shown in Table 1 (Figure 5e, Table S23). Differential methylation at 66 (15.0%) *blood CpGs* associated with changes in nine traits, namely total body fat percentage (24 CpGs), BMI (27 CpGs), WC (32 CpGs), HDL cholesterol levels (2 CpGs) and size (18 CpGs), interleukin‐6 (3 CpGs), leptin (19 CpGs), systolic blood pressure (2 CpGs), and fasting insulin (5 CpGs), showing that despite the size of these smaller methylomic responses and the lack of support for *cis* regulatory effects, they were still able to mark improvements in immunometabolic health.

### 2.9 Chronological age predictors have increased in accuracy but still capture more than just the passage of time

In the field of epigenetics and aging, several algorithms have been developed to predict chronological age (cAge) from DNAm data. Here, we predicted cAge changes following the GOTO intervention by applying three such clocks (namely Horvath, (2013), Zhang et al. (2019), and Bernabeu et al. (2023)) to DNAm from pre‐ and post‐intervention blood samples. Correlations between actual age, which increased by 13 weeks across this longitudinal study, and predicted cAge were then calculated (Table S24). Predictions of cAge using Horvath, one of the original epigenetic clocks, were moderately correlated with actual age (*r* = 0.730, *p*_FDR_ = 7.6 × 10^−34^), showing that from its inception these algorithms have performed well.

Looking at more recent clocks, we observe increases in accuracy over time with the Zhang (*r* = 0.888, *p*_FDR_ = 2.7 × 10^−67^) and Bernabeu (*r* = 0.923, *p*_FDR_ = 8.6 × 10^−82^) cAge predictions both correlating remarkably strongly with actual age at visit date. When looking at the predicted change in chronological age over this 13 week intervention, however, all three clocks calculated a reduction in age ranging from a 22.3 week decrease over the intervention (*p*_FDR_ = 0.200) returned by the original Horvath clock (Horvath, 2013), to a 12.5 week reduction (*p*_FDR_ = 0.155) predicted by the most recent Bernabeu cAge algorithm (Bernabeu et al., 2023). Overall, these results show that chronological age predictors are well correlated with and increasingly in line with actual age but considering that participants increased in age by 13 weeks over the intervention, they still have considerable residuals in some cases. This could indicate that such clocks are swayed by other factors, such as health improvements, and still need some refinement in order to capture only the passage of time.

### 2.10 GrimAge captures the effect of the GOTO intervention and associates with metabolic and physiological health improvements

**FIGURE 6:**
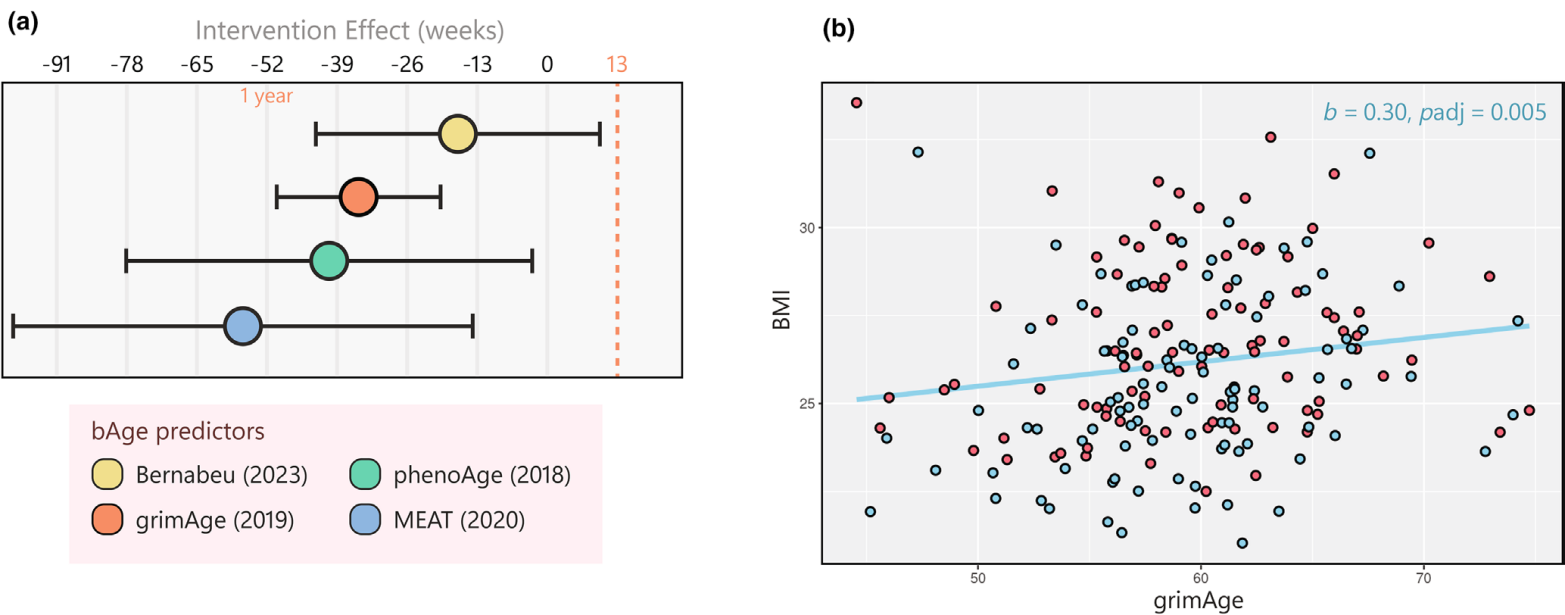
Characterization of the GOTO effects on measures of epigenetic age (a) Predicted decreases in biological age following GOTO for four modern algorithms, and (b) scatter plot showing the associations between grimAge and observed BMI both before (red) and after (blue) the GOTO intervention.

Recent biological age (bAge) predictors are commonly trained on a combination of age, health parameters, and mortality data. We investigated four such recent bAge clocks (namely Bernabeu et al. (2023), grimAge (Lu et al., 2019), phenoAge (Levine et al., 2018), and MEAT (Voisin et al., 2020)). The first three of these algorithms were trained using blood samples, and therefore we predicted bAge using DNAm from blood taken before and after the GOTO intervention. In contrast, MEAT is a muscle‐specific algorithm and so was applied to DNAm data from the GOTO muscle samples instead. Using a paired analysis, we estimated the effect of the GOTO intervention on biological age, adjusting for age, sex, and technical covariates (Figure 6; Table S25).

For all four clocks, bAge was predicted to decrease following the GOTO intervention with estimates ranging from a 16.1 week decrease as predicted by Bernabeu (*p*_FDR_ = 2.4 × 10^−1^) in blood to a 57.9 week decrease as predicted by the muscle‐specific MEAT algorithm (*p*_FDR_ = 2.1 × 10^−2^), with the majority of predicted reductions here being greater than estimated cAge decreases. After adjusting for multiple testing, however, we found that only the decreases in bAge predicted by grimAge (*β* = −34.6 weeks, *p*_FDR_ = 1.4 × 10^−4^) and MEAT (*β* = −57.9 weeks, *p*_FDR_ = 2.1 × 10^−2^) remained significant at the 5% level. As we saw the strongest methylation response to GOTO in muscle, these results support the development of tissue‐specific algorithms for the prediction of biological age. Additionally, since grimAge was reported to be more strongly associated with frailty risk as compared with other epigenetic age measures (Lieke et al., 2022), this finding highlights differences in developed algorithms and suggests a specific relevance of grimAge to aging populations.

To explore whether the observed reductions in bAge following the GOTO intervention were associated with immunometabolic health improvements, we performed paired analyses to calculate the association between changes in clocks with significant effects and 10 metabolic health parameters shown in Table 1 (Table S26). The directions of the observed associations supported grimAge and MEAT reductions as being linked to increases in metabolic health, with both measures having a negative association with adiponectin and positive associations with BMI, WC, leptin, IL‐6, and total body fat percentage. Seven of the 10 tested traits significantly associated with grimAge at the 5% level after adjusting for multiple testing, including BMI (*β* = 0.30, *p*_FDR_ = 5.2 × 10^−3^), total body fat percentage (*β* = 0.10, p_FDR_ = 3.9 × 10^−2^), and leptin (*β* = 0.08, *p*_FDR_ = 1.3 × 10^−2^), all of which were also associated with genome‐wide DNAm responses to GOTO in blood (Figure 6b). Taken together, these results demonstrate the power of blood based DNAm markers and bAge algorithms to capture health improvements, in particular in older adults.

## 3 DISCUSSION

### 3.1 Tissue‐specific DNA methylation responses potentially relate to key regulatory changes in muscle, adipose, and blood

Following a 13‐week combined lifestyle intervention, we observed DNA methylation (DNAm) responses at 162 CpGs in skeletal muscle, 230 CpGs in subcutaneous adipose tissue (SAT), and 441 CpGs in fasted blood. We characterized the regulatory nature of these CpGs, finding enrichments for active chromatin states and relevant transcription factor binding sites (TFBS). Then, by using gene expression and health parameter data from the same individuals, we performed multi‐omic analyses and uncovered relationships between epigenetic changes and metabolic health, with links to insulin sensitivity, regeneration potential, and body composition.

On a molecular level, the directions of DNAm responses were as expected from previously observed effects of this intervention on gene expression (Bogaards et al., 2024). In skeletal muscle, the CpGs predominantly decreased in methylation, aligning both with known effects of increasing physical activity on the muscle methylome (Plaza‐Diaz et al., 2022) and the genome‐wide increases in gene expression previously seen in muscle from this study. In contrast, there were increases in methylation at CpGs identified in SAT, tying in with global decreases in gene expression observed in this tissue after the GOTO intervention. Lastly, in blood, the signal was small in both the methylome and the transcriptome (Bogaards et al., 2024; Gehrmann et al., 2021), possibly due to molecular changes in blood lying further from functionally responding tissues.

In addition to linking our findings to previous genome‐wide transcriptome investigations, we also utilized the available RNA‐Seq data to investigate *cis* associations between differential gene expression and DNAm in the three tissues. In skeletal muscle, expression changes at 71 genes in close proximity to identified CpGs were associated with differential methylation. In contrast, 23 genes were linked to DNAm changes in SAT and there were only three genes with evidence of clear *cis* associations between genes and CpGs in blood. Looking at the function of the genes identified in muscle and SAT, many were responsible for insulin sensitivity and glucose uptake in relevant cell types. This included *GRB10* (Edick et al., 2020; Holt et al., 2018), which directly binds to and regulates insulin receptors, *PLEKHG4* (Ha & Lee, 2020; Machin et al., 2021; Shrestha et al., 2021), which is implicated in the translocation of GLUT4 transporters to the membrane in skeletal muscle cells, and *DMRT3* (Pujar et al., 2019), an insulin sensitivity marker specific to subcutaneous adipose tissues. In particular, the lowered expression of *GRB10* observed here is known to enhance insulin induced PI3K/Akt signalling and glucose uptake in myotubes and increase muscle size (Holt et al., 2012, 2018; Mokbel et al., 2014; Plaza‐Diaz et al., 2022). Observed changes in fasting insulin levels were associated with DNAm responses in all three tissues (muscle: 2 CpGs, SAT: 3 CpGs; blood: 5 CpGs), although there was no overlap with the relevant genes. As caloric restriction and exercise have established effects on insulin resistance (Dubé et al., 2011; Johnson et al., 2016) with consequences for immunometabolic health (Roberts et al., 2013), this finding illustrates potential molecular mechanisms behind these effects.

We also observed enrichments for regulatory chromatin states, such as enhancers and regions flanking active transcription start sites (TSS), in our muscle and blood CpGs, and relevant TFBS were enriched in sequences within 50 bp of the differentially methylated CpGs in all three tissues. In muscle, we identified Fos and JunB binding sites in proximity of our CpGs, and these transcription factors are known to be important for muscle health (Puntschart et al., 1998), and in SAT, there was enrichment for binding sites of four known GATA family TFs, established as involved in the initial stages of adipogenesis and obesity (Tong et al., 2000). Since the main mechanism by which DNAm is thought to elicit functional effects on nearby expression is by modulating the accessibility of regulatory sites for transcription factors (Kaluscha et al., 2022), these findings strengthen the plausibility that DNAm changes at the identified CpGs are regulatory and may have functional effects on expression of the associated nearby DEGs.

### 3.2 Differential methylation is linked to metabolic health improvements and decreases in estimated epigenetic age

Since the Growing Old Together (GOTO) intervention imparted a metabolic health benefit on the study population (Beekman et al., 2020; van de Rest et al., 2016), we investigated how these improvements associated with identified changes in DNAm. At over half of the muscle CpGs and almost a third of the adipose CpGs, DNAm responses were associated with changes in one or more health parameters. For example, DNAm in all three tissues was associated with not only fasting insulin but also a total of eight of the 10 tested traits, including total body fat percentage (blood: 24 CpGs, adipose: 39 CpGs, muscle: 13 CpGs), leptin (blood: 19 CpGs, adipose: 4 CpGs, muscle: 14 CpGs), and BMI (blood: 27 CpGs, adipose: 12 CpGs, muscle: 7 CpGs). Favorable changes in body composition, as seen in the GOTO intervention study, are associated with a more balanced secretion of adipokines from adipose tissue, reducing the risk of insulin resistance and type 2 diabetes. We show here that DNAm responses in relevant tissues are associated with these key measures of immunometabolic risk.

Previous investigations into the health benefits imparted by this intervention have demonstrated sex‐specific effects, possibly due to both physiological differences between sexes and the personalized nature of the protocol. For example, sex‐specific differences in muscle performance are partly attributed to larger proportions of type‐I fibres in women and characterized by slow oxidative metabolism (Haizlip et al., 2015). When looking at the transcriptomic responses to the GOTO intervention, previous studies were able to identify these sex‐specific responses (Bogaards et al., 2024). However, in this study where we investigated effects at over 750 thousand CpGs across the genome, we had insufficient power to consider the two sexes separately. In the future, it will be important to uncover the molecular mechanisms behind observed disparities between men and women by performing larger intervention studies that are adequately powered for stratification by sex.

Lastly, we calculated chronological (cAge) and biological (bAge) age in our blood and muscle samples using available methylome algorithms (Bernabeu et al., 2023; Horvath, 2013; Levine et al., 2018; Lu et al., 2019; Voisin et al., 2020; Zhang et al., 2019). These demonstrated the impressive precision and accuracy of current epigenetic clocks for calendar age prediction, with estimates from the most recent model (Bernabeu et al., 2023) being highly correlated with actual age (*r* = 0.923). However, all tested cAge algorithms still supported a decrease in age following the intervention despite participants actually aging 13 weeks, making it plausible that these estimates are still influenced by other factors such as metabolic health improvements.

To investigate this further, we evaluated if four predictors of biological age (bAge) could capture the health benefits of the GOTO intervention. All four algorithms predicted a decrease in bAge following the GOTO intervention ranging from −16.1 weeks to −57.9 weeks, larger than the previous cAge estimates. The muscle‐specific MEAT algorithm represented the greatest effect and it, alongside reductions in bAge as predicted by grimAge, were still significant at the 5% level after adjusting for multiple testing. GrimAge, in particular, was associated with observed improvements in seven of the 10 tested health parameters, including BMI, circulating leptin levels, and total body fat percentage, with all three also associated with DNAm in all tissues. GrimAge and the metabolomics‐based score, MetaboHealth, have both previously been reported as good measures of health improvements (Bogaards et al., 2024), frailty (Lieke et al., 2022), and mortality (Deelen et al., 2019; Lu et al., 2019). The beneficial shifts observed here in these scores indicate potentially global and long‐term health improvements from the GOTO intervention protocol, and also highlight the possible value of molecular algorithms such as these for monitoring effects of interventions in general, and specifically in older populations.

It is important to note that this intervention was carried out in healthy, older adults. For older individuals, for example with a risk of sarcopenia, this mild intervention may not be the most optimal regime. Other interventions, including ProMuscle and a novel upcoming study VOILA, are better focused on improving muscle mass and strength by including resistance training, increased protein intake, or protein supplementation (Leenders et al., 2013; van Dongen et al., 2017). Clinical study populations of older individuals may also require other response markers due to the higher levels of acute inflammatory proteins in population‐based elderly.

Overall, our in‐depth study of the methylome, transcriptome, and phenome exemplifies, the biological changes that older adults experience following a mild intervention, such as GOTO. The absence of any overlap between the identified sets of CpGs demonstrates the strong tissue‐specificity of these findings and this, coupled with the distinct directional differences (hypermethylation in adipose tissue and hypomethylation in muscle), highlights the importance of using a multi‐tissue approach when investigating the influence of environmental changes on the methylome. As DNAm is only one type of epigenetic modification, more in‐depth interpretation of these findings may require examination of other layers of the epigenome, such as chromatin accessibility using ATAC‐seq, and larger sample sizes would enable explorations of how DNAm in various tissues influences metabolic health and examination of any sex‐specific responses.

## 4 CONCLUSIONS

This study established the methylomic responses to a 13‐week lifestyle intervention in older adults in both circulating cells and relevant metabolic tissues. We identified differential methylation at CpGs located in regulatory regions in close proximity to transcription factor binding sites. Effects at these CpGs were associated with differential expression of genes important for insulin sensitivity, including *GRB10* in muscle and *DMRT3* in adipose, and with imparted metabolic health benefits. Identified loci may be investigated to monitor immunometabolic risk, progression of disease, and response to treatment in the future. The GOTO response was also represented by four epigenomic biological age markers and GrimAge, in particular, was able to capture the health improvements imparted to the participants. This study further demonstrates the importance of collecting biologically relevant tissues in intervention studies and highlights how modifiable molecular markers can capture health improvements following lifestyle changes in older people.

## 5 METHODS

### 5.1 Recruitment

The GOTO study is nested within the Leiden Longevity Study (LLS), a longitudinal cohort of long‐lived Caucasian siblings, their offspring, and partners thereof. The GOTO study (van de Rest et al., 2016) recruited healthy, older (mean age 63 years) adults (*N* = 164) between June 2012 and April 2013. Individuals between 46 and 75 years with a BMI between 23 and 35 kg/m (Knowler et al., 2002) from the pool of offspring and partners were eligible for the study.

Exclusion criteria included being on diabetic medication (for type 1 or 2 diabetes), having high fasting blood glucose levels (≥7.0 mmol/L), recent weight change (≥3 kg in the past 6 months), engagement in heavy or intensive physical activity (top sport or physically heavy work), any disease or condition that seriously affects body weight and/or body composition, recent immobilization (for over 1 week in the last 3 months), psychiatric or behavioral problems, use of thyroid medication or immunosuppressive drugs, concurrent participation in any other intervention studies or weight management programs, or not being registered with a general practitioner.

### 5.2 Intervention protocol

Expanding on the combined lifestyle arm of the CALERIE study (Kraus et al., 2019), GOTO participants reduced their energy balance by 25% for 13 weeks, through a combination of caloric restriction and increased physical activity. Informed by baseline questionnaires on energy intake (150‐item FFQ) and expenditure (IPAQ‐SF), dieticians and physiotherapists prescribed individual guidelines to achieve the intervention. Participants were advised to increase physical activity in a way that was compatible with their lifestyle, and dietary guidelines aimed to follow the “Dutch Guidelines for a Healthy Diet” (2006).

To check and stimulate adherence to the intervention, there was weekly contact with both the dietician and physiotherapist. Participants recorded their adherence to the intervention plan in a diary, and two 24‐h recalls were performed during the first and last month of the intervention. Days of the recall were unannounced to the participants and randomized to obtain a good distribution of weekdays and weekends. During monthly home visits, body weight and composition were measured.

### 5.3 Sample collection

Both prior and post intervention, blood (95 mL) was drawn by venepuncture between 8 and 9 a.m. in the hospital after at least 10 h of fasting. The participants consumed a SLM Nutridrink TM72 representative of a typical Northern European meal (300 kcal: 35% energy from fat, 50% from carbohydrate, and 16% protein) between 9 a.m. and noon on the same day. Following this, skeletal muscle biopsies were taken from the musculus vastus lateralis and a subcutaneous adipose biopsy was taken from the abdomen. Biopsies were taken under local anaesthetic and immediately frozen in liquid nitrogen before being stored at −80°C for subsequent analysis.

Of the 164 individuals in the GOTO study, we profiled DNAm from 104 individuals at both timepoints for multiple tissues (sample *n* = 562). All 562 samples represented distinct samples from a unique timepoint, individual, and tissue combination and not duplicates. DNA from whole blood (*n* = 206) was isolated using QIAmp DNA Mini kits (QIAgen) and using NucleoMag Tissue kits (Machery Nagel) for adipose (*n* = 188) and muscle (*n* = 168) samples. Pairs of samples were shuffled and plated so that they would be adjacent on the same array. These pairs were randomized across eight 96‐well plates by tissue, age, and sex using Omixer (Sinke et al., 2021), and sent for profiling using the Infinium MethylationEPIC Kit (Illumina, Helmholtz Institute).

### 5.4 DNA methylation data

Following receipt of the methylation data as. IDAT files, preprocessing and QC followed the DNAmArray pipeline (Sinke et al., 2019). MethylAid (van Iterson et al., 2014) plots were used to visualize and check sample quality. Due to technical issues with three of the Infinium MethylationEPIC arrays, 25 samples failed quality control checks. These alongside their pairs (*n* = 30) were removed from the data, and 24 samples with sufficient remaining material were reprofiled and subsequently passed QC checks.

After combining data from both waves, tissue identity was confirmed with PCA plots, and four outlying samples and their pairs (*n* = 8) were removed. Sample mismatches were detected and resolved by comparing genotype data with DNAm‐derived genotypes using omicsPrint (van Iterson et al., 2018). Individuals (*n* = 6) identified from diary data as non‐compliers were also removed. Lastly, methylation‐predicted sex was used as a final check of sample identity.

The data underwent functional normalization using four PCs, followed by removal of outlying or unreliable values, such as those based on low bead number (0.20%), intensity (0.08%), that were not distinguishable from background noise (0.37%), or more than 3 IQR from the nearest quartile per CpG (0.28%). Any probes or samples with over 5% missingness were removed (0.00% of samples, 1.06% of CpGs). Additionally, cross‐reactive, polymorphic (Zhou et al., 2016), poorly reproducible (Sugden et al., 2020), blacklisted (Amemiya et al., 2019), and sex chromosomal probes were removed. The resulting dataset contained DNA methylation data at 755,777 CpGs from 534 samples (196 blood, 178 adipose, and 160 muscle) from 102 individuals (mean age 63 years). For 66 individuals (64.7%), we had complete data from all three tissues at both timepoints.

### 5.5 RNA sequencing data

RNA isolation and sequencing has been described previously (Bogaards et al., 2024). In short, libraries were prepared using Illumina TruSeq version 2 library preparation kits. Data processing was performed using the in‐house BIOPET Gentrap pipeline (Zhernakova et al., 2017). The following steps were part of the data processing: low quality trimming using sickle version 12.00. Cutadapt version 1.1 was used to perform the adapter clipping. The reads were aligned to GRCh37 while masking for SNPs common in the Dutch population (GoNL 45 MAF > 0.01), using STAR version 2.3.0e. Picard version 2.4.1. was used to perform sam to bam conversion and sorting. Read quantification was performed using htseq‐count version 0.6.1.p1 using Ensembl gene annotations version 86 for gene definitions. In blood, the sequencing resulted in an average of 37.2 million reads per sample, 97% (±0.4%) of which were mapped. In SAT, samples had an average of 11.4 million sequenced reads, 95% (±1.6%) of which were mapped. In muscle, an average of 36.9 million sequence reads per sample, 98% (±0.4%) of which were mapped.

### 5.6 Cell type proportion data

In muscle, we predicted cell types using the MuSiC algorithm (Wang et al., 2019), which has been shown to outperform other methods in its characterization of cellular heterogeneity in complex tissues when provided with an appropriate reference (Avila Cobos et al., 2020). Using publicly available single nuclei expression data, we predicted muscle cell‐type proportions from bulk expression data (Perez et al., 2022). For fat samples, no suitable scRNA reference atlas was available, and therefore we utilized CIBERSORTx (Newman et al., 2019), the successor of one of the highest performing bulk deconvolution methods (Avila Cobos et al., 2020). We input a publicly available signature matrix which has previously predicted cell types from bulk adipose tissue RNAseq in TwinsUK and GTEx (Glastonbury et al., 2019).

In fasted blood, the percentages of cell types (neutrophils, lymphocytes, monocytes, eosinophils, and basophils) were measured with a blood differential test. To investigate the intervention effect on specific subtypes, we also predicted blood cell type proportions. Using an scRNA reference atlas (Xie et al., 2020) combined with whole blood expression data, the MuSiC algorithm (Wang et al., 2019) estimated 32 different cell types. Furthermore, the IDOL (Koestler et al., 2016) and IDOL extended (Salas et al., 2022) algorithms predicted six and 12 subtypes from the DNA methylation data, respectively.

### 5.7 Statistical analysis

To evaluate the effect of the intervention on DNAm, we performed a mixed model test with a fixed effect for the intervention (time) and an individual random effect (ID), adjusting for confounders (age, sex, and smoking), technical covariates (plate and array row), and the first five PCs. The Bioconductor package bacon (van Iterson et al., 2017) was used to inspect and adjust for bias and inflation of the test statistics, using default priors (*α* = 1.28, *β* = 0.36). For all models, estimates of inflation and bias were used to identify any anomalies in the data. *p* values were adjusted for multiple testing using the FDR method.

$$\begin{array}{c} {\text{DNAm}}_{\mathrm{ijk}}\sim {\text{time}}_{\mathrm{ij}}+{\mathrm{age}}_{i}+{\mathrm{sex}}_{i}+{\text{smoke}}_{i}+\text{plate}1_{\mathrm{ij}}+\ldots +\text{plate}10_{\mathrm{ij}}\\ \;+{\text{arrayRow}}_{\mathrm{ij}}+\mathrm{PC}1_{\mathrm{ij}}+\ldots +\mathrm{PC}5_{\mathrm{ij}}+\left(1|{\mathrm{ID}}_{i}\right) \end{array}$$

In muscle, we additionally adjusted for predicted endothelial nuclei proportions. These models were fit for each tissue individually using the limma package in R.

$$\begin{array}{c} {\text{DNAm}}_{\mathrm{ijk}}\sim {\text{time}}_{\mathrm{ij}}+{\mathrm{age}}_{i}+{\mathrm{sex}}_{i}+{\text{smoke}}_{i}+\text{plate}1_{\mathrm{ij}}+\ldots +\text{plate}10_{\mathrm{ij}}\\ \;+{\text{arrayRow}}_{\mathrm{ij}}+{\text{endo}}_{\mathrm{ij}}+\mathrm{PC}1_{\mathrm{ij}}+\ldots +\mathrm{PC}5_{\mathrm{ij}}+\left(1|{\mathrm{ID}}_{i}\right) \end{array}$$

### 5.8 CpG interpretation

#### 5.8.1 Differentially methylated regions

To assess the number of distinct genomic loci in our results, differentially methylated regions (DMRs) were identified using the DMRfinder algorithm (Slieker et al., 2013) as implemented in the DNAmArray workflow (Sinke et al., 2019). DMRs were defined as regions with at least three differentially methylated positions (DMPs) with an inter‐CpG distance of less than 1 kb, allowing a maximum of three non‐DMPs across a DMR. Next, the number of distinct loci were calculated as the total number of DMPs minus the number of DMPs in DMRs plus the number of DMRs called by DMRfinder.

#### 5.8.2 Chromatin state enrichment analyses

FDR significant CpGs were annotated to chromatin state using an appropriate Roadmap reference epigenome (E062 for blood, E063 for adipose, E107 and E108 for muscle) (Kundaje et al., 2015). Logistic regressions models were fit using the glm function in R to calculate and test odds ratios (ORs) of significance for each of the 15 chromatin states. Nominal *p* values were adjusted for multiple testing using FDR and enrichments or depletions were identified at the 5% significance threshold.

#### 5.8.3 Transcription factor‐binding site (TFBS) enrichment analyses

A 50 bp window around significant CpGs was scanned using findMotifsGenome.pl. from HOMER (Heinz et al., 2010) for enrichment of known motifs compared to background noise. ENCODE TFBS annotation for 171 TFs and CpGs on the EPIC array (Zhou et al., 2016) was used to further investigate the size of binding sites and distance from CpG to summit. TFs associated with enriched TFBS were examined for links with pathways specific to the tissue in which the enrichment was found.

#### 5.8.4 Gene annotation

Genomic locations of human transcripts, exons, CDS, and genes were imported from the Ensembl database using makeTxDbFromEnsembl from the GenomicFeatures Bioconductor package (Lawrence et al., 2013). These were used to annotate each of our CpGs to their nearest gene, in addition to saving a list of all genes which lay within 100 kb of each CpG.

#### 5.8.5 Differential gene expression

Gene expression changes were analysed as described previously but with both sexes combined in a single analysis (Bogaards et al., 2024). Briefly, the differential gene expression analysis was performed using linear mixed models in limma in combination with VOOM normalization. *p* values were adjusted for multiple testing using the FDR method and assessed at the 5% significance level. Models were adjusted for technical factors, age, and sex as fixed effects and included a random effect for ID.

#### 5.8.6 eQTM analyses

Using GOTO gene expression data from each tissue, we investigated the association between DNA methylation at our set of CpGs and expression levels of genes within 100 kb. Expression data was filtered for genes with low levels of expression and the edgeR Bioconductor package was used to calculate log2 CPM (Robinson et al., 2010). Lastly, RIN transformations were applied for each gene as described previously (Bonder et al., 2017).

$$\begin{array}{c} {\text{DNAm}}_{\mathrm{ij}}\sim {\text{gene}}_{\mathrm{ij}}+{\mathrm{age}}_{i}+{\mathrm{sex}}_{i}+{\text{smoke}}_{i}+\text{plate}1_{\mathrm{ij}}+\ldots +\text{plate}10_{\mathrm{ij}}\\ \;+{\text{arrayRow}}_{\mathrm{ij}}+{\text{flowcell}}_{\mathrm{ij}}+\left(1|{\mathrm{ID}}_{i}\right) \end{array}$$

Nominal *p* values were adjusted for multiple testing using FDR and assessed at the 5% significance level.

#### 5.8.7 Gene set enrichment analyses (GSEA)

Using a list of genes whose expression changed with nearby methylation as input, we performed GSEA. The associated gene names were then used as input for GSEA. We used recent (updated in the last 5 years) databases relating to human health and disease downloaded from Enrichr (GO Biological Process 2023, KEGG Human 2021, and Reactome 2022). These were imported into R and analyses were performed using the enrichr function from clusterProfiler (Xu et al., 2024). *p* values were adjusted for multiple testing using FDR and significance was assessed at the 5% level.

#### 5.8.8 Health parameter associations

We investigated the association between DNA methylation at our set of CpGs and 10 health parameters, adjusting for age, sex, smoking, technical covariates, and with a random effect for ID.

$$\begin{array}{c} {\text{DNAm}}_{\mathrm{ij}}\sim {\text{trait}}_{\mathrm{ij}}+{\mathrm{age}}_{i}+{\mathrm{sex}}_{i}+{\text{smoke}}_{i}+\text{plate}1_{\mathrm{ij}}+\ldots +\text{plate}10_{\mathrm{ij}}\\ \;+{\text{arrayRow}}_{\mathrm{ij}}+\left(1|{\mathrm{ID}}_{i}\right) \end{array}$$

For each trait, nominal *p* values were adjusted for multiple testing using FDR and assessed at the 5% significance level.

### 5.9 Epigenetic clock algorithms

#### 5.9.1 Chronological age prediction

Using the dnaMethyAge package (Wang et al., 2023), we predicted Horvath, (2013), Zhang et al. (2019), and Bernabeu et al. (2023) chronological ages (cAges). Correlations between these estimates and actual age was assessed using a Pearson's correlation test with the cor.test function in R. Coefficients, 95% confidence intervals, and *p* values were saved and further adjusted for multiple testing using FDR. Significance was assessed at the 5% level.

#### 5.9.2 Biological age predictions

Using the dnaMethyAge package (Wang et al., 2023), we predicted LevineM2018 (PhenoAge) (Levine et al., 2018). grimAge was estimated using the coefficients, R, and Python scripts provided by the researchers who developed this measure (Lu et al., 2019). Bernabeu's bAge (Bernabeu et al., 2023) was predicted by combining grimAge, DNAm data, and phenotype data in the bage_predictor.R script provided on their GitHub (elenabernabeu/cage_bage). Lastly, MEAT biological ages (Voisin et al., 2020) were predicted from muscle DNAm data using the MEAT Bioconductor package for R.

For all four bAge predictions, paired analyses were used to estimate how they changed following the GOTO intervention, adjusting for age, sex, technical covariates and with a random effect for ID:

$${\text{bAge}}_{\mathrm{ij}}\sim {\text{time}}_{i}+{\mathrm{age}}_{i}+{\mathrm{sex}}_{i}+\text{plate}1_{\mathrm{ij}}+\ldots +\text{plate}10_{\mathrm{ij}}+{\text{arrayRow}}_{\mathrm{ij}}+\left(1|{\mathrm{ID}}_{i}\right)$$

Nominal *p* values were adjusted for multiple testing using FDR and assessed at the 5% significance level.

#### 5.9.3 Associations between grimAge and metabolic health parameters

We investigated the association between grimAge as predicted from our blood DNAm data and 10 health parameters using a paired analysis, adjusting for age, sex, technical covariates, and with a random effect for ID.

$${\text{grimAge}}_{\mathrm{ij}}\sim {\text{trait}}_{\mathrm{ij}}+{\mathrm{age}}_{i}+{\mathrm{sex}}_{i}+\text{plate}1_{\mathrm{ij}}+\ldots +\text{plate}10_{\mathrm{ij}}+{\text{arrayRow}}_{\mathrm{ij}}+\left(1|{\mathrm{ID}}_{i}\right)$$

For each trait, nominal *p* values were adjusted for multiple testing using FDR and assessed at the 5% significance level.

## AUTHOR CONTRIBUTIONS

P.E.S. and C.d.G. designed the study. M.B. collected and curated the health data. N.L. and E.S. generated the DNA methylation and transcriptome data. L.S., B.T.H., M.B., and P.E.S. designed the data analysis approach, L.S. and F.A.B. performed data analysis, I.M. and L.S. performed the cell deconvolution analyses, D.B. provided expertise on epigenetic clocks, Y.R., A.B., G.B‐B., and C.T. provided expertise on muscle physiology, M.W. profiled DNAm in the three tissues and provided vital intellectual contributions, and L.S., Y.R., A.B., G.B‐B., C.T., B.T.H., P.E.S., and M.B. performed the research and interpreted the data. All authors were involved in drafting and revising the manuscript.

## FUNDING INFORMATION

The research leading to these results was supported by the Joint Programming Initiative “a Healthy Diet for a Healthy Life” (JPI‐HDHL) DIMENSION project (ZonMW project number: 529051021) and ZonMW Project VOILA (ZonMW project number: 457001001). The underlying intervention study was financially supported by the Netherlands Consortium for Healthy Ageing (grant 050‐060‐810), in the framework of the Netherlands Genomics Initiative, Netherlands Organization for Scientific Research (NWO) and by BBMRI‐NL, a Research Infrastructure financed by the Dutch government (NWO 184.021.007). The funding agencies had no role in the design and conduct of the study; collection, management, analysis, and interpretation of the data; and preparation, review, or approval of the manuscript.

## CONFLICT OF INTEREST STATEMENT

The authors declare that they have no competing interests.

## CONSENT FOR PUBLICATION

Not applicable for this study. No individual data, such as individual details, images, or videos are included in this manuscript.

## Supporting information


Figure S1.

Tables S1–S26.

## Data Availability

The data underlying the findings in this paper can be found in the Tables S1–S26. All other related data supporting the findings of this study are accessible upon reasonable request to the corresponding author. The individual‐level data are not publicly available due to privacy or ethical restrictions. All other data used in this study are publicly available: reference epigenome data is available from ROADMAP (Kundaje et al., 2015) and TFBS data is available within the HOMER software (Heinz et al., 2010). All the software and programmes used to conduct these analyses are freely available. Scripts used during this analysis are available on GitHub at https://nebulyra.github.io/goto_dnam/.
