# Supplementary material for: Tissue‐specific methylomic responses to a lifestyle intervention in older adults associate with metabolic and physiological health improvements
Source: Aging Cell. 2024 Dec 1;24(4):e14431. doi: 10.1111/acel.14431 (PMC11984676; doi:10.1111/acel.14431)

**Supplementary Figure 1:** Barplots of effect sizes in the three tissues for significant CpGs in each tissue (along the x-axis, ordered by effect size). Bars are coloured by p-values for the relevant tissue model (muscle for row 1, adipose for row 2, and blood for row 3), highlighting tissue-specific effects.

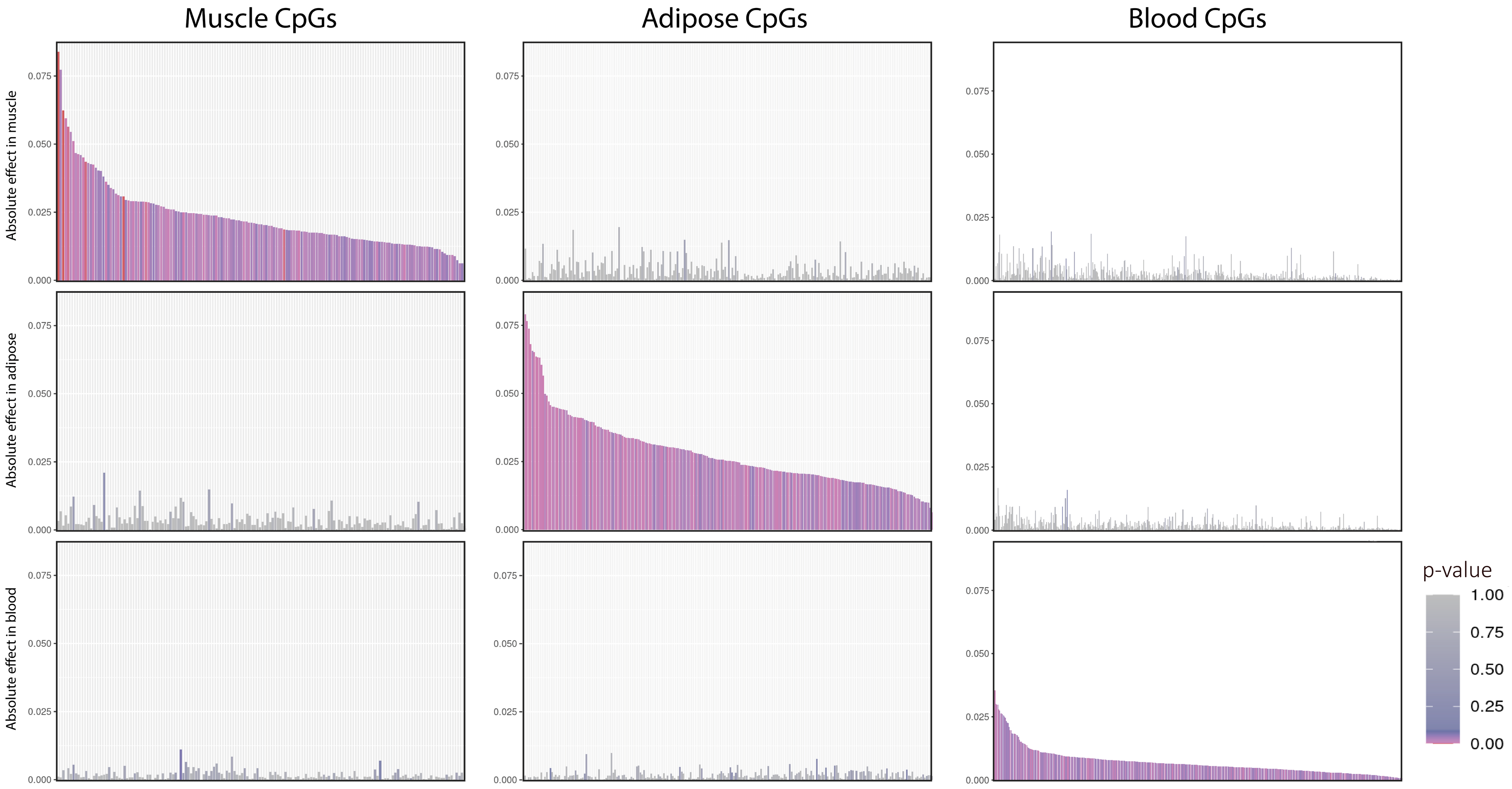

Supplement: Supplementary file 1 — Figure S1. [file ACEL-24-e14431-s001.pdf]
